# Supplementary material for: Influence of nutritional supplements on antibody levels in pregnant women vaccinated with inactivated SARS-CoV-2 vaccines
Source: PLoS One. 2024 Mar 7;19(3):e0289255. doi: 10.1371/journal.pone.0289255 (PMC10919710; doi:10.1371/journal.pone.0289255)
Supplement: S1 File — (DOCX) [file pone.0289255.s001.docx]

**S1 Table. Demographic characteristics of the participants taking folic acid.**

| **Characteristics** | **Total（N=825）** | **Neutralizing Antibody** | | ***P*** | **IgG** | | ***P*** |
| --- | --- | --- | --- | --- | --- | --- | --- |
|  |  | **+**  **（N=365）** | **-**  **（N=460）** |  | **+**  **（N=384）** | **-**  **（N=441）** |  |
| **Age group, y** |  |  |  | 0.185 |  |  | 0.025* |
| <35 | 730（88.5） | 329（90.1） | 401（87.2） |  | 350（91.1） | 380（86.2） |  |
| ≥35 | 95（11.5） | 36（9.9） | 59（12.8） |  | 34（8.9） | 61（13.8） |  |
| **BMI***^a^* |  |  |  | 0.983 |  |  | 0.419 |
| <18.5 | 160（19.4） | 70（19.2） | 90（19.6） |  | 68（17.7） | 92（20.9） |  |
| 18.5-24 | 508（61.6） | 226（61.9） | 282（61.3） |  | 245（63.8） | 263（59.6） |  |
| 24-28 | 128（15.5） | 51（14.0） | 77（16.7） |  | 55（14.3） | 73（16.6） |  |
| ≥28 | 29（3.5） | 18（4.9） | 11（2.4） |  | 16（4.2） | 13（2.9） |  |
| **Gravidity** |  |  |  | 0.734 |  |  | 0.411 |
| 1 | 345（41.8） | 158（43.3） | 187（40.7） |  | 170（44.3） | 175（39.7） |  |
| 2 | 320（38.8） | 137（37.5） | 183（39.8） |  | 143（37.2） | 177（40.1） |  |
| ≥3 | 160（19.4） | 70（19.2） | 90（19.6） |  | 71（18.5） | 89（20.2） |  |
| **Birth** |  |  |  | 0.920 |  |  | 0.572 |
| 0 | 405（49.1） | 182（49.9） | 223（48.5） |  | 196（51.0） | 209（47.4） |  |
| 1 | 318（38.5） | 139（38.1） | 179（38.9） |  | 143（37.2） | 175（39.7） |  |
| ≥2 | 102（12.4） | 44（12.1） | 58（12.6） |  | 45（11.7） | 57（12.9） |  |
| **Education** |  |  |  | 0.829 |  |  | 0.909 |
| Middle school and below | 198（24.0） | 86（23.6） | 112（24.3） |  | 91（23.7） | 107（24.3） |  |
| High school | 176（21.3） | 83（22.7） | 93（20.2） |  | 86（22.4） | 90（20.4） |  |
| Junior college | 286（34.7） | 126（34.5） | 160（34.8） |  | 130（33.9） | 156（35.4） |  |
| Bachelor and above | 165（20.0） | 70（19.2） | 95（20.7） |  | 77（20.1） | 88（20.0） |  |
| **Household income per capita, m** |  |  |  | 0.097 |  |  | 0.266 |
| <4000 | 129（15.6） | 57（15.6） | 72（15.7） |  | 65（16.9） | 64（14.5） |  |
| 4000~6000 | 248（30.1） | 111（30.4） | 137（29.8） |  | 119（31.0） | 129（29.3） |  |
| 6000~10000 | 285（34.5） | 138（37.8） | 147（32.0） |  | 135（35.2） | 150（34.0） |  |
| ≥10000 | 163（19.8） | 59（16.2） | 104（22.6） |  | 65（16.9） | 98（22.2） |  |
| **Smoking** |  |  |  | 1.000 |  |  | 1.000 |
| No | 819（99.3） | 362（99.2） | 457（99.3） |  | 381（99.2） | 438（99.3） |  |
| Yes | 6（0.7） | 3（0.8） | 3（0.7） |  | 3（0.8） | 3（0.7） |  |
| **Number of exercises per week** |  |  |  | 0.285 |  |  | 0.652 |
| 0 | 312（37.8） | 149（40.8） | 163（35.4） |  | 151（39.3） | 161（36.5） |  |
| <3 | 52（6.3） | 22（6.0） | 30（6.5） |  | 25（6.5） | 27（6.1） |  |
| ≥3 | 461（55.9） | 194（53.2） | 267（58.0） |  | 208（54.2） | 253（57.4） |  |
| **Adverse events** |  |  |  | 0.111 |  |  | 0.027* |
| No | 455（55.2） | 190（52.1） | 265（57.6） |  | 196（51.0） | 259（58.7） |  |
| Yes | 370（44.8） | 175（47.9） | 195（42.4） |  | 188（49.0） | 182（41.3） |  |

**S1 Table. (Continued)**

| **Characteristics** | **Total（N=825）** | **Neutralizing Antibody** | | | ***P*** | **IgG** | | ***P*** |
| --- | --- | --- | --- | --- | --- | --- | --- | --- |
|  |  | **+**  **（N=365）** | **-**  **（N=460）** | |  | **+**  **（N=384）** | **-**  **（N=441）** |  |
| **Doses of vaccination** |  |  | |  | <0.001* |  |  | <0.001* |
| One dose | 79（9.6） | 5（1.4） | | 74（16.1） |  | 5（1.3） | 74（16.8） |  |
| Two doses | 633（76.7） | 248（67.9） | | 385（83.7） |  | 266（69.3） | 367（83.2） |  |
| Three doses | 113（13.7） | 112（30.7） | | 1（0.2） |  | 113（29.4） | 0（0.0） |  |
| **Inoculation durations, wk** |  |  | |  | <0.001* |  |  | <0.001* |
| <20 | 109（13.2） | 88（24.1） | | 21（4.6） |  | 97（25.3） | 12（2.7） |  |
| 20-24 | 98（11.9） | 47（12.9） | | 51（11.1） |  | 53（13.8） | 45（10.2） |  |
| 24-28 | 188（22.8） | 86（23.6） | | 102（22.2） |  | 84（21.9） | 104（23.6） |  |
| 28-32 | 221（26.8） | 73（20.0） | | 148（32.2） |  | 83（21.6） | 138（31.3） |  |
| 32-36 | 133（16.1） | 44（12.1） | | 89（19.3） |  | 44（11.5） | 89（20.2） |  |
| ≥36 | 76（9.2） | 27（7.4） | | 49（10.7） |  | 23（6.0） | 53（12.0） |  |

*****: *P* < 0.05

**S2 Table. Demographic characteristics of the participants taking iron supplements.**

| **Characteristics** | **Total（N=165）** | **Neutralizing Antibody** | | ***P*** | **IgG** | | ***P*** |
| --- | --- | --- | --- | --- | --- | --- | --- |
|  |  | **+**  **（N=60）** | **-**  **（N=105）** |  | **+**  **（N=56）** | **-**  **（N=109）** |  |
| **Age group, y** |  |  |  | 0.900 |  |  | 0.593 |
| <35 | 141（85.5） | 51（85.0） | 90（85.7） |  | 49（87.5） | 92（84.4） |  |
| ≥35 | 24（14.5） | 9（15.0） | 15（14.3） |  | 7（12.5） | 17（15.6） |  |
| **BMI***^a^* |  |  |  | 0.096 |  |  | 0.300 |
| <18.5 | 30（18.2） | 6（10.0） | 24（22.9） |  | 7（12.5） | 23（21.1） |  |
| 18.5-24 | 104（63.0） | 40（66.7） | 64（61.0） |  | 36（64.3） | 68（62.4） |  |
| 24-28 | 24（14.5） | 11（18.3） | 13（12.4） |  | 11（19.6） | 13（11.9） |  |
| ≥28 | 7（4.3） | 3（5.0） | 4（3.8） |  | 2（3.6） | 5（4.6） |  |
| **Gravidity** |  |  |  | 0.665 |  |  | 0.628 |
| 1 | 68（41.2） | 26（43.3） | 42（40.0） |  | 23（41.1） | 45（41.3） |  |
| 2 | 65（39.4） | 21（35.0） | 44（41.9） |  | 20（35.7） | 45（41.3） |  |
| ≥3 | 32（19.4） | 13（21.7） | 19（18.1） |  | 13（23.2） | 19（17.4） |  |
| **Birth** |  |  |  | 0.489 |  |  | 0.966 |
| 0 | 77（46.7） | 29（48.3） | 48（45.7） |  | 26（46.4） | 51（46.8） |  |
| 1 | 66（40.0） | 21（35.0） | 45（42.9） |  | 22（39.3） | 44（40.4） |  |
| ≥2 | 22（13.3） | 10（16.7） | 12（11.4） |  | 8（14.3） | 14（12.8） |  |
| **Education** |  |  |  | 0.537 |  |  | 0.723 |
| Middle school and below | 40（24.2） | 16（26.7） | 24（22.9） |  | 15（26.8） | 25（22.9） |  |
| High school | 38（23.0） | 10（16.7） | 28（26.7） |  | 10（17.9） | 28（25.7） |  |
| Junior college | 48（29.1） | 19（31.7） | 29（27.6） |  | 17（30.4） | 31（28.4） |  |
| Bachelor and above | 39（23.6） | 15（25.0） | 24（22.9） |  | 14（25.0） | 25（22.9） |  |
| **Household income per capita, m** |  |  |  | 0.181 |  |  | 0.363 |
| <4000 | 23（13.9） | 6（10.0） | 17（16.2） |  | 5（8.9） | 18（16.5） |  |
| 4000~6000 | 50（30.3） | 14（23.3） | 36（34.3） |  | 16（28.6） | 34（31.2） |  |
| 6000~10000 | 58（35.2） | 24（40.0） | 34（32.4） |  | 20（35.7） | 38（34.9） |  |
| ≥10000 | 34（20.6） | 16（26.7） | 18（17.1） |  | 15（26.8） | 19（17.4） |  |
| **Smoking** |  |  |  | 0.355 |  |  | 0.211 |
| No | 160（97.0） | 57（95.0） | 103（98.1） |  | 53（94.6） | 107（98.2） |  |
| Yes | 5（3.0） | 3（5.0） | 2（1.9） |  | 3（5.4） | 2（1.8） |  |
| **Number of exercises per week** |  |  |  | 0.918 |  |  | 0.514 |
| 0 | 39（23.6） | 13（21.7） | 26（24.8） |  | 11（19.6） | 28（25.7） |  |
| <3 | 9（5.5） | 3（5.0） | 6（5.7） |  | 2（3.6） | 7（6.4） |  |
| ≥3 | 117（70.9） | 44（73.3） | 73（69.5） |  | 43（76.8） | 74（67.9） |  |
| **Adverse events** |  |  |  | 0.358 |  |  | 0.604 |
| No | 93（56.4） | 31（51.7） | 62（59.0） |  | 30（53.6） | 63（57.8） |  |
| Yes | 72（43.6） | 29（48.3） | 43（41.0） |  | 26（46.4） | 46（42.2） |  |

**S2 Table. (Continued)**

| **Characteristics** | **Total（N=165）** | **Neutralizing Antibody** | | | ***P*** | **IgG** | | ***P*** |
| --- | --- | --- | --- | --- | --- | --- | --- | --- |
|  |  | **+**  **（N=60）** | | **-**  **（N=105）** |  | **+**  **（N=56）** | **-**  **（N=109）** |  |
| **Doses of vaccination** |  |  |  | | <0.001* |  |  | <0.001* |
| One dose | 41（24.8） | 3（5.0） | 38（36.2） | |  | 2（3.6） | 39（35.8） |  |
| Two doses | 114（69.1） | 47（78.3） | 67（63.8） | |  | 44（78.6） | 70（64.2） |  |
| Three doses | 10（6.1） | 10（16.7） | 0（0.0） | |  | 10（17.9） | 0（0.0） |  |
| **Inoculation durations, wk** |  |  |  | | 0.063 |  |  | 0.208 |
| <20 | 5（3.0） | 4（6.7） | 1（1.0） | |  | 4（7.1） | 1（0.9） |  |
| 20-24 | 10（6.1） | 3（5.0） | 7（6.7） | |  | 5（8.9） | 5（4.6） |  |
| 24-28 | 30（18.2） | 15（25.0） | 15（14.3） | |  | 11（19.6） | 19（17.4） |  |
| 28-32 | 53（32.1） | 14（23.3） | 39（37.1） | |  | 14（25.0） | 39（35.8） |  |
| 32-36 | 48（29.1） | 15（25.0） | 33（31.4） | |  | 16（28.6） | 32（29.4） |  |
| ≥36 | 19（11.5） | 9（15.0） | 10（9.5） | |  | 6（10.7） | 13（11.9） |  |

*****: *P* < 0.05

**S3 Table. Demographic characteristics of the participants taking DHA.**

| **Characteristics** | **Total（N=197）** | **Neutralizing Antibody** | | ***P*** | **IgG** | | ***P*** |
| --- | --- | --- | --- | --- | --- | --- | --- |
|  |  | **+**  **（N=66）** | **-**  **（N=131）** |  | **+**  **（N=76）** | **-**  **（N=121）** |  |
| **Age group, y** |  |  |  | 0.690 |  |  | 0.294 |
| <35 | 167（84.8） | 55（83.3） | 112（85.5） |  | 67（88.2） | 100（82.6） |  |
| ≥35 | 30（15.2） | 11（16.7） | 19（14.5） |  | 9（11.8） | 21（17.4） |  |
| **BMI***^a^* |  |  |  | 0.826 |  |  | 0.975 |
| <18.5 | 35（17.8） | 11（16.7） | 24（18.3） |  | 14（18.4） | 21（17.4） |  |
| 18.5-24 | 122（61.9） | 40（60.6） | 82（62.6） |  | 47（61.8） | 75（62.0） |  |
| 24-28 | 31（15.7） | 11（16.7） | 20（15.3） |  | 11（14.4） | 20（16.6） |  |
| ≥28 | 9（4.6） | 4（6.0） | 5（3.8） |  | 4（5.3） | 5（4.1） |  |
| **Gravidity** |  |  |  | 0.461 |  |  | 0.040* |
| 1 | 85（43.1） | 29（43.9） | 56（42.7） |  | 35（46.1） | 50（41.3） |  |
| 2 | 81（41.1） | 24（36.4） | 57（43.5） |  | 24（31.6） | 57（471.） |  |
| ≥3 | 31（15.7） | 13（19.7） | 18（13.7） |  | 17（22.4） | 14（11.6） |  |
| **Birth** |  |  |  | 0.620 |  |  | 0.231 |
| 0 | 94（47.7） | 31（47.0） | 63（48.1） |  | 38（50.0） | 56（46.3） |  |
| 1 | 82（41.6） | 26（39.4） | 56（42.7） |  | 27（35.5） | 55（45.5） |  |
| ≥2 | 21（10.7） | 9（13.6） | 12（9.2） |  | 11（14.5） | 10（8.3） |  |
| **Education** |  |  |  | 0.786 |  |  | 0.734 |
| Middle school and below | 39（19.8） | 13（19.7） | 26（19.8） |  | 15（19.7） | 24（19.8） |  |
| High school | 43（21.8） | 12（18.2） | 31（23.7） |  | 14（18.4） | 29（24.0） |  |
| Junior college | 65（33.0） | 22（33.3） | 43（32.8） |  | 25（32.9） | 40（33.1） |  |
| Bachelor and above | 50（25.4） | 19（28.8） | 31（23.7） |  | 22（28.9） | 28（23.1） |  |
| **Household income per capita, m** |  |  |  | 0.582 |  |  | 0.696 |
| <4000 | 23（11.7） | 6（9.1） | 17（13.0） |  | 11（14.5） | 12（9.9） |  |
| 4000~6000 | 57（28.9） | 19（28.8） | 38（29.0） |  | 20（26.3） | 37（30.6） |  |
| 6000~10000 | 69（35.0） | 27（40.9） | 42（32.1） |  | 28（36.8） | 41（33.9） |  |
| ≥10000 | 48（24.4） | 14（21.1） | 34（26.0） |  | 17（22.4） | 31（25.6） |  |
| **Smoking** |  |  |  | 1.000 |  |  | 1.000 |
| No | 192（97.5） | 64（97.0） | 128（97.7） |  | 74（97.4） | 118（97.5） |  |
| Yes | 5（2.5） | 2（3.0） | 3（2.3） |  | 2（2.6） | 3（2.5） |  |
| **Number of exercises per week** |  |  |  | 0.710 |  |  | 0.312 |
| 0 | 59（29.9） | 19（28.8） | 40（30.5） |  | 19（25.0） | 40（33.1） |  |
| <3 | 11（5.6） | 5（7.6） | 6（4.6） |  | 6（7.9） | 5（4.1） |  |
| ≥3 | 127（64.5） | 42（63.6） | 85（64.9） |  | 51（67.1） | 76（62.8） |  |
| **Adverse events** |  |  |  | 0.875 |  |  | 0.988 |
| No | 109（55.3） | 36（54.5） | 73（55.7） |  | 42（55.3） | 67（55.4） |  |
| Yes | 88（44.7） | 30（45.5） | 58（44.3） |  | 34（44.7） | 54（44.6） |  |

**S3 Table. (Continued)**

| **Characteristics** | **Total（N=197）** | **Neutralizing Antibody** | | ***P*** | **IgG** | | ***P*** |
| --- | --- | --- | --- | --- | --- | --- | --- |
|  |  | **+**  **（N=66）** | **-**  **（N=131）** |  | **+**  **（N=76）** | **-**  **（N=121）** |  |
| **Doses of vaccination** |  |  |  | <0.001* |  |  | <0.001* |
| One dose | 30（15.2） | 2（3.0） | 28（21.4） |  | 3（3.9） | 27（22.3） |  |
| Two doses | 154（78.2） | 51（77.3） | 103（78.6） |  | 60（78.9） | 94（77.7） |  |
| Three doses | 13（6.6） | 13（19.7） | 0（0.0） |  | 13（17.1） | 0（0.0） |  |
| **Inoculation durations, wk** |  |  |  | 0.009* |  |  | <0.001* |
| <20 | 8（4.1） | 3（4.5） | 5（3.8） |  | 7（9.2） | 1（0.8） |  |
| 20-24 | 22（11.2） | 13（19.7） | 9（6.9） |  | 14（18.4） | 8（6.6） |  |
| 24-28 | 50（25.4） | 22（33.3） | 28（21.4） |  | 25（32.9） | 25（20.7） |  |
| 28-32 | 61（31.0） | 12（18.2） | 49（37.4） |  | 14（18.4） | 47（38.8） |  |
| 32-36 | 38（19.3） | 10（15.2） | 28（21.4） |  | 12（15.8） | 26（21.5） |  |
| ≥36 | 18（9.1） | 6（9.1） | 12（9.2） |  | 4（5.3） | 14（11.6） |  |

*****: *P* < 0.05
