# Supplementary material for: Influence of nutritional supplements on antibody levels in pregnant women vaccinated with inactivated SARS-CoV-2 vaccines
Source: PLoS One. 2024 Mar 7;19(3):e0289255. doi: 10.1371/journal.pone.0289255 (PMC10919710; doi:10.1371/journal.pone.0289255)
Supplement: Attachment — Submitted filename: Response to Reviewers.docx [file pone.0289255.s003.docx]

[Au](javascript:;)g 13, 2023

Dear Editor,

Thank you for providing us this opportunity to revise our manuscript entitled “Effects of nutritional supplements on antibody levels in pregnant women vaccinated with inactivated SARS-CoV-2 vaccines” (Manuscript ID: PONE-D-23-21116). On behalf of my colleagues, I am submitting our revised manuscript. We appreciate the reviewers’ and editor’s positive and insightful comments. We have carefully considered all the comments and revised the manuscript accordingly. In order to facilitate the review process, we provided a point-by-point response to each of the comments. The precise page and line in the revised manuscript where each change was made in response to the comments were provided as well.

We appreciated for Editors and Reviewers’ warm work earnestly. We have tried our best to improve and revise the manuscript. We hope that the manuscript is now acceptable. Should you have any additional requests or questions, please do not hesitate to contact me.

We look forward to hearing from you.

**RESPONSES TO THE REVIEWER#1’ S COMMENTS**

**Comment 1:** **The meaning of the reported P values in Table 1 and Table 3 is unclear. For example, if the P value in line "Age group" (Table 1, 0.054) indicates that there are no significant differences between the folic acid group and non-folic acid group in terms of age, then how should we interpret the P value in line "Doses of vaccination" (Table 3, <0.001)? It suggests that there is a significant difference between the neutralizing antibody positive group and neutralizing antibody negative group in terms of vaccination doses. However, in lines 201-203, the authors conclude that "For pregnant women who took folic acid, the positive rate of SARS-CoV-2 neutralizing antibody varied significantly with vaccine doses (P < 0.001) and time since vaccination (P < 0.001)." Such inconsistencies are present throughout the article. If the P values have different implications in different tables, the authors should provide clear explanations in the manuscript.**

**Response:** Thanks for the reviewer's comment. As you mentioned above，the P value in line "Doses of vaccination" (Table 3, <0.001) suggests that there is a significant difference between the neutralizing antibody positive group and neutralizing antibody negative group in terms of vaccination doses. I'm sorry for not expressing clearly.We have revised (show in line 212-226) as:

For pregnant women who took folic acid, there was a significant difference between the neutralizing antibody positive group and neutralizing antibody negative group in terms of vaccination doses (*P* < 0.001) and time since vaccination (*P* < 0.001). There was a significant difference between IgG antibody negative and positive groups in terms of age (*P* = 0.025), adverse events (*P* = 0.027), vaccination doses (*P* < 0.001), and time since vaccination (*P* < 0.001). Among pregnant women who took iron, there was a significant difference between the negative and positive groups of neutralizing antibody, as well as the negative and positive groups of IgG antibody, in terms of vaccination doses (*P* < 0.001). In pregnant women taking DHA, there was a significant difference between IgG antibody negative and positive groups in terms of gravidity *(P* = 0.040), vaccination doses (*P* < 0.001), and time since vaccination (*P* < 0.001). There was a statistically significant difference between the neutralizing antibody positive group and neutralizing antibody negative group in terms of vaccination doses (*P* < 0.001) and time since vaccination (*P* = 0.009) .

**Comment 2:** **In lines 186-187, the authors state "We found significant differences in gravidity (P = 0.002), parity (P = 0.012), and education (P = 0.008) according to whether the pregnant women took folic acid." The intended meaning of this statement is unclear. Similar sentences also appear on lines 190-193.**

**Response:** Thanks for the reviewer's comment. We have revised (show in line 196-198,200-204) as:

We found that there were statistically significant differences between groups of pregnant women who did or did not take folic acid in terms of gravidity (*P* = 0.002), parity (*P* = 0.012), and education (*P* = 0.008).

There were statistically significant differences between groups of pregnant women who did or did not take iron supplements in terms of smoking (*P* = 0.008), weekly exercise frequency (*P* < 0.001), vaccine doses (*P* < 0.001), time since vaccination (*P* < 0.001), SARS-CoV-2 neutralizing antibody (*P* = 0.017), and IgG antibody (*P* < 0.001) .

**Comment 3:In Table 6, the authors adjusted for variables such as age, BMI, education, number of exercises per week, adverse events, inoculation durations, and doses of vaccination. The rationale behind selecting these variables and not others is not explained. Additionally, the manuscript lacks univariate analysis of these variables in relation to antibody levels.**

**Response:** Thanks for the reviewer's comment. We have revised (show in line 167-173) as:

The adjusted factors were determined through the results of univariate analysis and literature review. Model 1 was adjusted for age, BMI, education, gravidity, parity, adverse events, inoculation durations, doses of vaccination. Model 2 was adjusted for age, BMI, smoking, number of exercises per week, adverse events, inoculation durations, doses of vaccination. Model 3 was adjusted for age, BMI, smoking, number of exercises per week, adverse events, inoculation durations, doses of vaccination.

**RESPONSES TO THE REVIEWER#2’ S COMMENTS**

**Comment 4:** Page 5, add citation for“Pregnant women with iron deficiency anemia are more susceptible to SARS-CoV-2 infection.”

**Response:** Thanks for the reviewer's comment.We have added citation for the word.

**Comment 5:** Page 5, in the last paragraph of the introduction, please show how this study add to the literature.

**Response:** Thanks for the reviewer's comment.We have revised (show in line 113-115) as:

This study can provide a certain reference for the question of the influence of women taking nutrients during pregnancy on the level of antibody produced by COVID-19 vaccines.

**Comment 6:** Page 5, add a flowchart for the study participants.

**Response:** Thanks for the reviewer's comment.We have added a flowchart in page 6.

**Comment 7:** Page 6, in the study variable section, try to make clear that which variables are outcome variables, exposure variables, and covariates, respective.

**Response:** Thanks for the reviewer's comment.We have revised (show in line 140-143) as:

The presence of nutrient supplements was the exposure variable, negative or positive antibody level were outcome variables, and age, BMI, parity, education and income level, smoking, number of exercises per week, doses of vaccination, adverse reactions, and time since vaccination were covariates.

**Comment 8:** Page 7, justify why fisher test are needed.

**Response:** Thanks for the reviewer's comment.We have revised (show in line 164) as:

When the sample size n < 40 or the expected frequency T < 1 use Fisher's test.

**Comment 9:** Page 12, line 200, authors further analyzed the general demographic characteristics of the pregnant women taking the three nutritional supplements separately. Tables 3-5 can be combined to one Table. Also, these tables seem not quite contribute well to the study aim, which can be considered to be the supplemental Table.

**Response:** Thanks for the reviewer's comment.We have revised.

**Comment 10:** Page 20, page 283, it is better to use "multivariable analysis", rather than "multivariate analysis".

**Response:** Thanks for the reviewer's comment.We have revised (show in line 230) .

**Comment 11:** Page 21, Table 6 is the final model to answer the study aim. In bivariate analyses, the associations between exposure variables (e.g., iron supplements and DHA) and outcome variables seems to be statistically significant but the associations disappeared in the adjusted models. Any reasons? For each model based on three separate exposure variables, whether authors adjusted for different confounding factors (e.g., see Table 1)? What is the definition of the confounder? Also, are there any effect modifiers? In the statistic analysis section, authors can provide more details.

**Response:** Thanks for the reviewer's comment.We have revised (show in line 167-173) as:

The adjusted factors were determined through the results of univariate analysis and literature review. Model 1 was adjusted for age, BMI, education, gravidity, parity, adverse events, inoculation durations, doses of vaccination. Model 2 was adjusted for age, BMI, smoking, number of exercises per week, adverse events, inoculation durations, doses of vaccination. Model 3 was adjusted for age, BMI, smoking, number of exercises per week, adverse events, inoculation durations, doses of vaccination.

**Comment 12:** Page 21, in discussion section, any previous studies in China or worldwide have been done? Are their findings consistent with the present study? Try to add some novelty/strength of this study.

**Response:** Thanks for the reviewer's comment.We have revised (show in line 335-338) as:

The strength of this study is that it is the first to examine the influence of nutrient supplements taken during pregnancy on the antibody level produced by pregnant women who have received COVID-19 vaccines. The finding of this study provides a certain degree of reference for pregnant women in this regard.
